# Antidiabetic and Antihyperlipidemic Effects of Methanolic Extract of Leaves of *Spondias mombin* in Streptozotocin-Induced Diabetic Rats

**DOI:** 10.3389/fphys.2022.870399

**Published:** 2022-05-10

**Authors:** Ramachawolran Gobinath, Subramani Parasuraman, Subramaniam Sreeramanan, Balaji Enugutti, Suresh V. Chinni

**Affiliations:** ^1^ Department of Biotechnology, Faculty of Applied Sciences, AIMST University, Bedong, Malaysia; ^2^ Department of Foundation, RCSI & UCD Malaysia Campus, Georgetown, Malaysia; ^3^ Department of Pharmacology, Faculty of Pharmacy, AIMST University, Bedong, Malaysia; ^4^ Department of Industrial Biotechnology, Universiti Sains Malaysia, Georgetown, Malaysia; ^5^ Centre for Chemical Biology, Universiti Sains Malaysia (USM), Bayan Lepas, Malaysia; ^6^ National Poison Centre, Universiti Sains Malaysia (USM), Penang, Malaysia; ^7^ Gregor Mendel Institute (GMI), Austrian Academy of Sciences, Vienna Biocenter (VBC), Vienna, Austria

**Keywords:** antidiabetic, antihyperlipidemic, *Spondias mombin*, steptozotocin, diabetes

## Abstract

**Objective:** *Spondias mombin* is a plant that reported to have anticonvulsant, antimicrobial, antioxidant, antiulcer, antiasthmatic, and wound healing activities. Diabetes dyslipidemic effect of *Spondias mombin* leaves is not clear. Hence, current study planned to evaluate the antidiabetic and antihyperlipidemic effects of methanolic extract of leaves of *Spondias mombin* (MESM) in streptozotocin (STZ) induced diabetic rats.

**Methods:** Phytochemicals were determined by standard method and antioxidant activity was determined by DPPH free radical scavenging and FRAP assay. Diabetes was induced by injecting a single dose of STZ (55 mg/kg) into female sprague dawley rats. After 3 days of induction of diabetes, the diabetic animals were treated for 28 days with MESM (125, 250, and 500 mg/kg) and glibenclamide (20 mg/kg) orally. The body weight of rats and blood glucose levels were monitored at regular intervals during the experiment. At the end of study, blood sample was collected from all the animals and subjected to biochemical, lipid profile, and they were sacrificed and their organs such as pancreas, liver and kidney were used for histopathological analysis.

**Results:** Quantitative analysis of MESM showed the presence of anthraquinone, tannins, saponins, steroid, phenols, flavonoids, alkaloids, and reducing sugars. Reduction in body weight and elevated blood glucose were observed in diabetic rats. Treatment with MESM in a concentration of 125, 250, and 500 mg/kg significantly reversed the elevated levels of blood glucose, reduced aspartate aminotransferase (AST), alanine aminotransferase (ALT), alkaline phosphatase (ALP), total bilirubin, urea, creatinine, total serum cholesterol (TC), serum triglyceride (TG), low-density lipoprotein (LDL), Very low-density lipoprotein (VLDL), and increased plasma insulin, total protein, albumin, globulin, A/G ratio, and high-density lipoprotein (HDL).

**Conclusion:** MESM exhibited a significant antidiabetic and antihyperlipidemic activities against STZ-induced diabetes in rats.

## 1 Introduction

Diabetes is a heterogeneous endocrine and metabolic illnesses characterised *via* hyperglycemia due to deficiency or diminished effectiveness of insulin action, insulin secretion or both. Hyperglycemia causes long-term health damage and failure of various organs particularly nerves, blood vessels, kidneys, eyes, and heart (Sneha and Gangil, 2019).

Diabetes mellitus can be classified into two main types, type 1 and type 2 (Roussel et al., 2021), with type 1 resulting from the body’s failure to produce insulin, and requires one to be injected with insulin (Smyth, 2021). Type 2 diabetes mellitus describes a condition of fasting hyperglycemia that occurs despite the availability of insulin (Effah-Yeboah et al., 2015). International Diabetic Federation (IDF) reported there are currently around 463 million people living with diabetes worldwide which is projected to increase to 700 million by 2045 (IDF Diabetes Atlas 9th edition 2019). Around 3.6 million people in Malaysia are confirmed with diabetes and seven million adults in Malaysia are predicted to get diabetes by end of 2025 (Institute for Public Health—NHMS 2019). This significant rise is due to Type 2 diabetes and consequence of excess body weight and physical inactivity. In type 2 diabetes mellitus, formation of Reactive Oxygen Species (ROS) resulted from impaired insulin synthesis due to pancreatic β-cell death by apoptosis. Hence, the body’s antioxidant system enhanced through usage of supplements and plant compounds which may reduce oxidative stress and prevents the disease at the beginning stage (Tangvarasittichai, 2015). Medicinal plants believed to provide efficient against health problems with minimum side effects with reasonable cost and easily available. Plant products may play a vital role to discover new therapeutic agents and gained recognition as origin of bioactive compounds such as hypoglycemic, antioxidants, and hypolipidemic agents (Zhang and Reddy, 2018).

*Spondias mombin* L. (Anarcadiaceae) is generally referred as “Hog plum” in English (Ezuruike and Prieto, 2014). In traditional medicine, *Spondias mombin* claimed as efficient in treating psychiatric disorders, duodenal disorders, gonorrhoea, inflammatory conditions, wounds, infections, diabetes, and to remove placenta during childbirth (Iwu, 1993). Decoction of the powdered flowers and leaves of *Spondias mombin* are used to reduce stomach pain, cystitis, urethritis, biliousness, enhance eye, and throat inflammation (Temitope et al., 2017). *Spondias mombin* roots and stem bark exhibited anti-inflammatory, antimicrobial, anti-mycobacterial, antiviral, hematinic, anthelmintic, and sedative activities (Nwidu et al., 2018). Antidiabetic and antihyperlipidemic activity of *Spondias mombin* is not clear. Hence, the present study is planned to evaluate the antidiabetic and antihyperlipidemic effect of methanolic extract of *Spondias mombin* (MESM) on streptozotocin (STZ)-induced diabetes mellitus in *Sprague dawley* rats.

## 2 Materials and Methods

### 2.1 Plant Material

Fresh and matured *Spondias mombin* leaves were collected from Semeling, Sungai Petani, Kedah. The plant was identified and authenticated by a botanist from herbarium department, (USM 11767/09/2018). The plant materials were washed; dried under the shade and pulse grinded using a grinding machine. The powder was kept in an air tight container for further use (Namani et al., 2016).

### 2.2 Chemicals

STZ was purchased from Sigma chemical company, Malaysia. All other solvents used in the experiments were purchased locally from Merck or SD fine Chemicals and were of analytical grade.

### 2.3 Extraction

*Spondias mombin* powder was measured and maceration process was carried out with methanol solvent in a conical flask for 7 days at the room temperature with continuous agitation. After maceration process completed, the leaves extract filtered using muslin cloth. The extract then dried by evaporation using rotary evaporator (Rotavapor^®^ R-210, BUCHI Corporation). The MESM was stored at −80°C until further use (Samuggam et al., 2021). The percentage yield of MESM is 91% w/w.

### 2.4 Phytochemical Screening

The MESM was tested for the presence of secondary metabolites like anthraquinone (hydrochloric acid and sulphuric acid), tannins (ferric chloride), saponins (distilled water), steroid (chloroform and acetic anhydride), phenols (ferric chloride), flavonoids (ammonia and sulphuric acid), alkaloids (hydrochloric acid and Dragendorff reagent), and reducing sugars (Fehling’s solution A and B) (Yadav and Agarwala, 2011; Sunitha et al., 2018).

### 2.5 Total Phenol and Flavonoid Content

Total phenolic content in methanol was measured using spectrophotometric method. 0.5 ml of plant sample added with 2.5 ml of 0.75% sodium carbonate solution and 2.5 ml of 1% Folin-Ciocalteu reagent added in a test tube. The mixture was incubated for 15 min at a temperature of 45°C and absorbance was measured at 765 nm. The standard calibration curve was plotted based on absorbance at 765 nm against concentration of gallic acid. Based on the calibration curve, total phenolic content was calculated and the results were expressed as gallic acid equivalent in mg/g (Kumari et al., 2016).

Total flavonoid content in methanol was measured using aluminium chloride colorimetric assay. 1 ml of plant sample added with 1 ml of standard quercetin solution, 4 ml of distilled water and 0.3 ml of 5% sodium nitrite solution followed by 2 ml of 1 M sodium hydroxide. The mixture was incubated for 15 min at a temperature of 45°C and absorbance was measured at 510 nm. The standard calibration curve was plotted using standard quercetin. The total flavonoid content was calculated from the calibration curve, and the results were expressed as quercetin equivalent in mg/g (Kumari et al., 2016).

### 2.6 Antioxidant Assay

#### 2.6.1 DPPH Free Radical Scavenging Assay

Radical scavenging activity was measured by method (Ayoola et al., 2008) with slight modification. Different concentrations of methanolic extract of *Spondias mombin* leaves (MESM) ranging from 0.5 to 3 mg/ml were prepared. The reaction mixture (3 ml) consists of 1 ml of DPPH solution (0.3 mM), l ml of the extract and 1 ml of methanol. The mixture was incubated for 30 min in dark and then the absorbance was measured at 517 nm. Quercetin was used as standard. A blank solution was prepared with DPPH and methanol.

The percentage of inhibition was calculated by using following equation

Percent inhibition = (A0—A1/A0) x 100

Where;

A0 = the absorbance of the blank

A1 = the absorbance of the sample.

The experiment was carried out in triplicate. Antioxidant activity was expressed as IC_50_ which is the concentration (ug/ml) of extract that is required for 50% of DPPH scavenging activity.

### 2.7 Ferric Reducing Antioxidant Power Assay

FRAP assay was performed according to the method (Muller et al., 2011) with slight modification. Different concentration of methanolic extract of *Spondias mombin* leaves at 5, 2.5, 1.25, 0.625, 0.312, 0.156 mg/ml were prepared each at volume of 100 µL and was mixed with 4.5 ml of FRAP reagent in test tubes, thoroughly mixed by vortexing and were incubated in water bath for 30 min at 37°C. The blank was prepared with FRAP working reagent and methanol. The aqueous solution of FeSO_4_.7H_2_O was used as standard. The absorbance of the samples was determined at 593 nm by UV spectrophotometer (UV-Vis Spectrophotometer, United States). The results were expressed as mg of ferrous equivalent per Gram of extract.

### 2.8 Experimental Animals

Adult female sprague dawley rats weight between 160 ± 20 g were purchased from (Universiti Sains Malaysia, Penang, Malaysia) and placed at 23 ± 2°C under 50 ± 5% humidity level for 12 h light and 12 h dark cycle. Rat pellet and water provided to the rats and acclimatised for 7 days before permitted to take part in the experiment. The study was approved by the AIMST University Human and Animal Ethics Committee (AUHAEC/FAS/2017/01) and the study was conducted according to the Animal Research Review Panel guidelines.

### 2.9 Acute Toxicity

Adult female sprague dawley rats were selected for the study. Acute toxicity study was conducted by applying fixed-dose procedure. Rats were subjected oral dose of *Spondias mombin* starting from 50, 100, 250, 500, 1,000, and 2000 mg/kg body weight (*n* = 3 per dose), respectively. Body weight, behaviour, autonomic profiles, and neurological changes were continuously monitored for 24 h (Parasuraman et al., 2015). Then, the animals were further monitored for 2 weeks for mortality accordance with the current guidelines of Organization for Economic Co-operation and Development (OECD) (Guideline, 2001).

### 2.10 Induction of Diabetes

Adult female sprague dawley rats were selected for the study. After acclimatization period of 1 week, single intraperitoneal injections of STZ (55 mg/kg) mixed with 0.05 M citrate buffer administered to experimentally induce diabetes mellitus (Lin et al., 2019). Twenty four hours later upon induction of diabetes mellitus, 5% w/v of glucose solution (2 ml/kg/BW) was orally given to the rats to avoid hypoglycemic mortality. Citrate buffer was intraperitoneally injected to the control rats. Few drops of blood sample was collected from tail vein 3 days after STZ injection to measure glucose levels using a glucometer (ACCU-CHEK^®^ Active, Roche Diagnostics, Mannheim, Germany). Rats with glucose levels >11 mmol/L were recognised as having diabetes and were used for the study.

### 2.11 Experimental Design

Adult female sprague dawley rats were divided into six groups each of six animals as follows:

Group I: Normal control
Group II: Diabetic control
Group III: Diabetic + glibenclamide (20 mg/kg)
Group IV: Diabetic + MESM (125 mg/kg)
Group V: Diabetic + MESM (250 mg/kg) Group VI: Diabetic + MESM (500 mg/kg)

The dose of glibenclamide was selected from the literature and the dose of *Spondias mombin* were selected from acute toxicity study findings (Parasuraman et al., 2019). Glibenclamide and MESM were suspended in 0.5% w/v carboxymethyl cellulose and administered orally. The animals in group I and II and were administered with 0.5% w/v carboxymethyl cellulose. Rats in group III were treated with 20 mg/kg body weight (BW) of glibenclamide (Gobinath et al., 2020). MESM at the dose levels of 125, 250, 500 mg/kg BW were administered to rats in group IV, V, and VII. On day 0, 7, 14, 21, and 28th day, few drops of blood samples were collected from tail vein to check the glucose levels using glucometer. Body weight variations were monitored on pre-study day, 14th day and 28th day for all the experimental animals. At the end of the study, blood samples were collected from the all the experimental animals, plasma were separated and used for the biochemical analysis.

### 2.12 Biochemical Analysis

At the 28th day, few millilitres (ml) of the blood sample were collected from tail vein in sample tubes containing EDTA. Biochemical parameter such as total plasma, total protein, glutamic oxaloacetic transaminase (AST), glutamic-pyruvic transaminase (ALT), and alkaline phosphatase (ALP), albumin, globulin, albumin/globulin (A/G) ratio, total bilirubin, urea, and creatinine and lipid parameter such as total cholesterol (TC), triglycerides (TG), low density lipoprotein (LDL), very low density lipoprotein (VLDL), high density lipoprotein (HDL), were estimated by “Cobas Integra 400 plus” analyzer.

### 2.13 Estimation of Plasma Insulin

Plasma insulin level was estimated using rat insulin enzyme linked immunosorbent assay (ELISA) kits (RayBiotech Inc., Norcross, GA, United States).

### 2.14 Histopathological Analysis

At the end of 28th day, experimental group rats were sacrificed and pancreas, liver, and kidney were isolated for hispathological analysis. Specimens sections were stored in formalin 10%. Tissue is cut at 5 µm section with Microtome (Thermo Electron Corporation, England) from paraffin-embedded tissue blocks. The sections was placed on a glass slide, de-paraffinized, rehydrated and stained with hematoxylin and eosin (H&E). After mounting with DPX and coverslip the slides were analysed under a light microscope and recorded.

### 2.15 Statistical Analysis

The mean ± standard error of the mean (SEM) values was calculated for each group. Statistical differences among the groups were determined using repeated measure ANOVA followed by Tukey’s *post hoc* test using SPSS. *p* < 0.05 was considered to be significant.

## 3 Results

### 3.1 Phytochemical Parameters on *Spondias mombin*

Phytochemical analysis on MESM showed the presence of anthraquinone, tannin, saponin, steroid, phenolic compounds, flavonoids, reducing sugar, and alkaloids as indicated in Table 1A.

**TABLE 1:** Effect of oral administration of methanolic extract of leaves of *Spondias mombin* (MESM) on sprague dawley rats.

**A) Phytochemical parameters**													
**Types of tests**										**Result**			
Anthraquinone										**+**			
Tannin										**+**			
Saponin										**+**			
Steroid										**+**			
Phenolic compounds										**+**			
Flavonoids										**+**			
Alkaloids										**+**			
Reducing sugar										**+**			
**B) Total phenol and flavonoids content**													
**Types of tests**										**Concentration**			
Total phenols										124.71 ± 0.61 mg GAE/g			
Total flavonoids										89.37 ± 0.13 mg QE/g			
**C) Antioxidant assay**													
**Antioxidant activity assay**										**Concentration**			
*S.mombin* DPPH activity IC_50_										1.62 ± 0.05 mg/ml			
Quercetin DPPH activity IC_50_										34.52 ± 0.51 mg/ml			
*S.mombin* FRAP assay										541.63 ± 1.53 mg FeSO_4_E/g			
**D) Effect of MESM on body weight (g)**													
					**Pre-study day**					**14th day**			**28th day**
Group I					175.88 ± 2.26					182.27 ± 1.88			191.77 ± 1.05
Group II					172.70 ± 1.36					162.19 ± 1.35***			150.94 ± 1.28***
Group III					174.04 ± 2.03					176.72 ± 2.01			187.44 ± 1.66
Group IV					170.71 ± 1.38					176.27 ± 0.87			188.09 ± 0.70
Group V					170.04 ± 2.14					177.44 ± 1.90			188.59 ± 1.91
Group VI					169.88 ± 2.46					178.60 ± 1.99			188.94 ± 1.22
**E) Effect of MESM on glucose level (mmol/L)**													
		**Pre-study day**		**7th day**			**14th day**		**21st day**			**28th day**	
Group I		5.60 ± 0.30		5.57 ± 0.35			5.28 ± 0.12		5.13 ± 0.26			5.22 ± 0.29	
Group II		17.45 ± 0.61***		17.12 ± 0.68			15.98 ± 0.54		16.02 ± 0.43			16.52 ± 0.45	
Group III		17.50 ± 0.61***		11.46 ± 0.57^###^			10.68 ± 0.38^###^		6.13 ± 0.11^###^			5.10 ± 0.09^###^	
Group IV		18.27 ± 0.49***		14.83 ± 0.85			14.32 ± 0.51		9.90 ± 0.30^###^			7.67 ± 0.21^###^	
Group V		17.85 ± 0.65***		13.75 ± 0.66^#^			12.78 ± 0.42^##^		9.22 ± 0.23^###^			6.10 ± 0.20^###^	
Group VI		17.82 ± 0.47***		12.02 ± 0.49^##^			12.00 ± 0.47^###^		8.88 ± 0.19^###^			5.38 ± 0.07^###^	
**F) Effect of MESM on biochemical parameters**													
	**Plasma insulin (μU/ml)**	**Total protein (g/dl)**	**AST (IU/L)**	**ALT (IU/L)**		**ALP (IU/L)**	**Albumin (g/dl)**	**Globulin (g/dl)**	**A/G ratio (g/dl)**		**Total bilirubin (mg/dl)**	**Urea (mg/dl)**	**Creatinine (mg/dl)**
Group I	19.08 ± 0.41	9.81 ± 0.35	46.15 ± 0.66	60.87 ± 0.37		121.18 ± 0.53	4.45 ± 0.17	3.46 ± 0.15	1.17 ± 0.02		0.35 ± 0.01	34.96 ± 0.57	0.79 ± 0.04
Group II	4.11 ± 0.17***	4.31 ± 0.38***	100.61 ± 1.05***	116.85 ± 0.95***		201.84 ± 0.54***	3.23 ± 0.18***	1.99 ± 0.10***	0.86 ± 0.02***		0.97 ± 0.02***	72.96 ± 0.61***	1.48 ± 0.09***
Group III	19.26 ± 0.36^###^	8.96 ± 0.41^###^	60.27 ± 0.66^###^	60.43 ± 0.54^###^		131.48 ± 0.70^###^	4.18 ± 0.18^###^	3.32 ± 0.08^###^	1.15 ± 0.028^###^		0.38 ± 0.02^###^	37.50 ± 0.48^###^	0.75 ± 0.06^###^
Group IV	7.77 ± 0.36^###^	5.60 ± 0.13^#^	98.19 ± 0.32	100.82 ± 1.24^###^		194.67 ± 0.75^###^	3.27 ± 0.10	2.33 ± 0.04	0.90 ± 0.02		0.91 ± 0.02	67.51 ± 0.26^###^	1.19 ± 0.04^##^
Group V	10.46 ± 0.39^###^	6.43 ± 0.16^###^	90.45 ± 0.44^###^	94.30 ± 0.33^###^		189.48 ± 0.37^###^	3.63 ± 0.08	2.45 ± 0.09^#^	0.95 ± 0.03		0.79 ± 0.03^###^	58.10 ± 0.32^###^	0.98 ± 0.02^###^
Group VI	14.33 ± 0.48###	7.92 ± 0.14###	79.18 ± 0.42^###^	85.02 ± 0.31^###^		163.17 ± 1.74^###^	3.92 ± 0.17^#^	2.89 ± 0.08^###^	1.12 ± 0.048^###^		0.55 ± 0.03^###^	44.10 ± 0.79^###^	0.89 ± 0.03^###^
**G) Effect of MESM on lipid profile (mg/dl)**													
		**TC**		**TG**			**HDL**		**LDL**			**VLDL**	
Group I		81.43 ± 0.46		101.97 ± 0.69			34.43 ± 0.75		43.72 ± 0.70			13.94 ± 0.28	
Group II		195.83 ± 0.65***		168.25 ± 0.99***			16.08 ± 0.45***		101.29 ± 2.07***			42.22 ± 0.49***	
Group III		94.12 ± 0.76^###^		81.81 ± 0.58^###^			37.95 ± 0.57^###^		41.26 ± 0.52^###^			13.75 ± 0.27^###^	
Group IV		185.19 ± 1.22^###^		163.26 ± 1.51			18.88 ± 0.31		79.91 ± 0.18^###^			37.91 ± 0.30^###^	
Group V		168.20 ± 1.18^###^		152.69 ± 1.24^###^			21.91 ± 0.61^###^		61.67 ± 0.46^###^			22.90 ± 0.56^###^	
Group VI		132.56 ± 0.82^###^		136.91 ± 1.42^###^			28.80 ± 2.06^###^		49.39 ± 0.40^###^			14.74 ± 0.25^###^	

AST, aspartate aminotransferase; ALT, alanine aminotransferase; ALP, alkaline phosphatase; TC, total serum cholesterol; TG, serum triglyceride; HDL, high-density lipoprotein; LDL, low-density lipoprotein; VLDL, very low-density lipoprotein.

All the values are Mean ± SEM (*n* = 6).

***p < 0.001 compare with control, ^#^ *p* < 0.05, ^##^ *p* < 0.01, and ^###^ *p* < 0.001, compare with diabetic control (Repeated measure ANOVA, followed by Tukey *post hoc* test).

### 3.2 Total Phenol and Flavonoid Content

Total phenolic and flavonoid analysis of MESM reported as 124.71 ± 0.61 mg GAE/g and 89.37 ± 0.13 mg QE/g respectively. Acute toxicity study in MESM treated rats did not exhibit any toxicological sign up to the dose of 2000 mg/kg. Hence, the antidiabetic effect of MESM was studied at the dose levels of 125, 250, and 500 mg/kg as indicated in Table 1B.

### 3.3 Antioxidant Activity

The IC_50_ of the MESM and quercetin in DPPH assay was 1.62 ± 0.05 mg/ml and 34.52 ± 0.51 mg/ml respectively and FRAP assay was 541.63 ± 1.53 mg FeSO_4_E/g as indicated in Table 1C.

### 3.4 Body Weight Analysis on Sprague Dawley Rats

The diabetic animals showed a significant reduction in the body weight from 14th day onwards when compared with that of control. Whereas, the animals treated with glibenclamide and MESM did not showed any reduction in body weight when compared with that of control (Table 1D).

### 3.5 Blood Glucose Analysis on Sprague Dawley Rats

Blood glucose levels after different oral doses of MESM presented in Table 1E. Rats in Group III treated with glibenclamide (20 mg/kg) showed significant decrease (*p* < 0.05) in blood glucose level compared with diabetic control rats from day 7 onwards. As expected in Group IV and Group V, mean blood glucose level significantly declined (*p* < 0.05) from day 21 onwards compared with diabetic control rats. Furthermore, Group VI rats had distinctly reduced blood glucose levels (*p* < 0.05) from day 14 onwards when compared with diabetic control.

### 3.6 Biochemical Parameters on Sprague Dawley Rats

Induction of diabetes caused significant increase in AST, ALT, ALP, total bilirubin, urea, and creatinine when compared with normal control. Furthermore, significant decrease in total protein, albumin, globulin and A/G ratio (*p* < 0.05) when compared with that of normal control (Table 1F). However, administration of different doses MESM to diabetic rats for 28 days recovered ALT, ALP, and urea from Group IV onwards, total protein, AST and total bilirubin from Group V onwards, globulin, A/G ratio, and albumin in Group VI compared with diabetic control rats.

### 3.7 Lipid Profile on Sprague Dawley Rats

The diabetes rats showed significant increase in TC, TG, LDL, and VLDL and decrease in HDL (*p* < 0.001) compared with normal control rats (Table 1G). However, administration of MESM caused significant decrease in TC, TG, LDL, VLDL, and increase in HDL (at 250 and 500 mg/kg) in comparison with control diabetic rats.

### 3.8 Plasma Insulin Level on Sprague Dawley Rats

Plasma insulin level was reduced significantly in diabetic rats while treatment with MESM increased plasma insulin level significantly (*p* < 0.001) at 125, 250, and 500 mg/kg dose (Table 1F).

### 3.9 Histopathological Analysis on Sprague Dawley Rats

**FIGURE 1:**
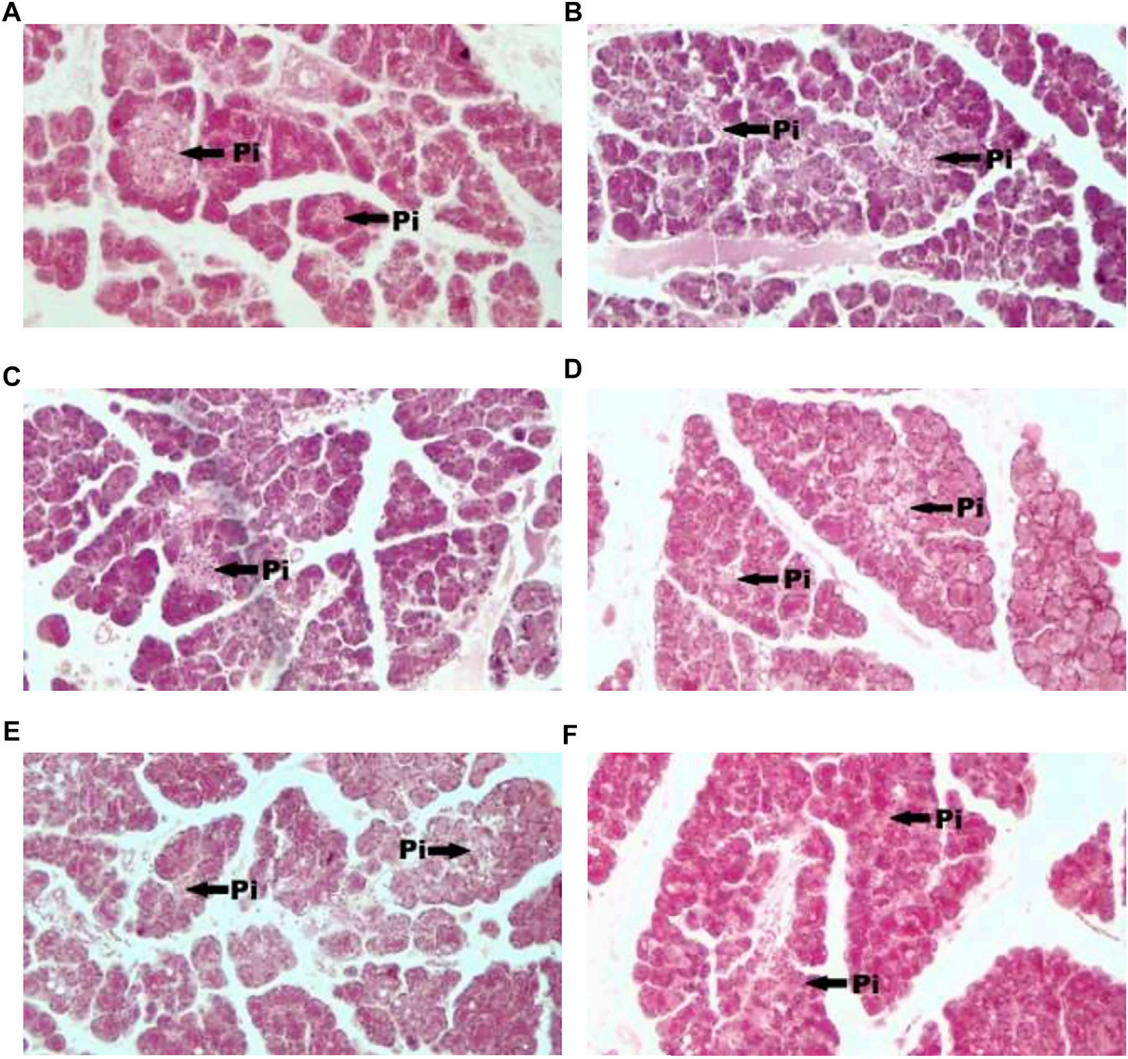
Photomicrograph of a section of pancreas of normal control **(A)**, diabetic control **(B)**, glibenclamide 20 mg/kg **(C)**, Spondias mombin 125 mg/kg **(D)**, Spondias mombin 250 mg/kg **(E)**, Spondias mombin 500 mg/kg **(F)**. PI-Pancreatic Islet. H&E 100X.

**FIGURE 2:**
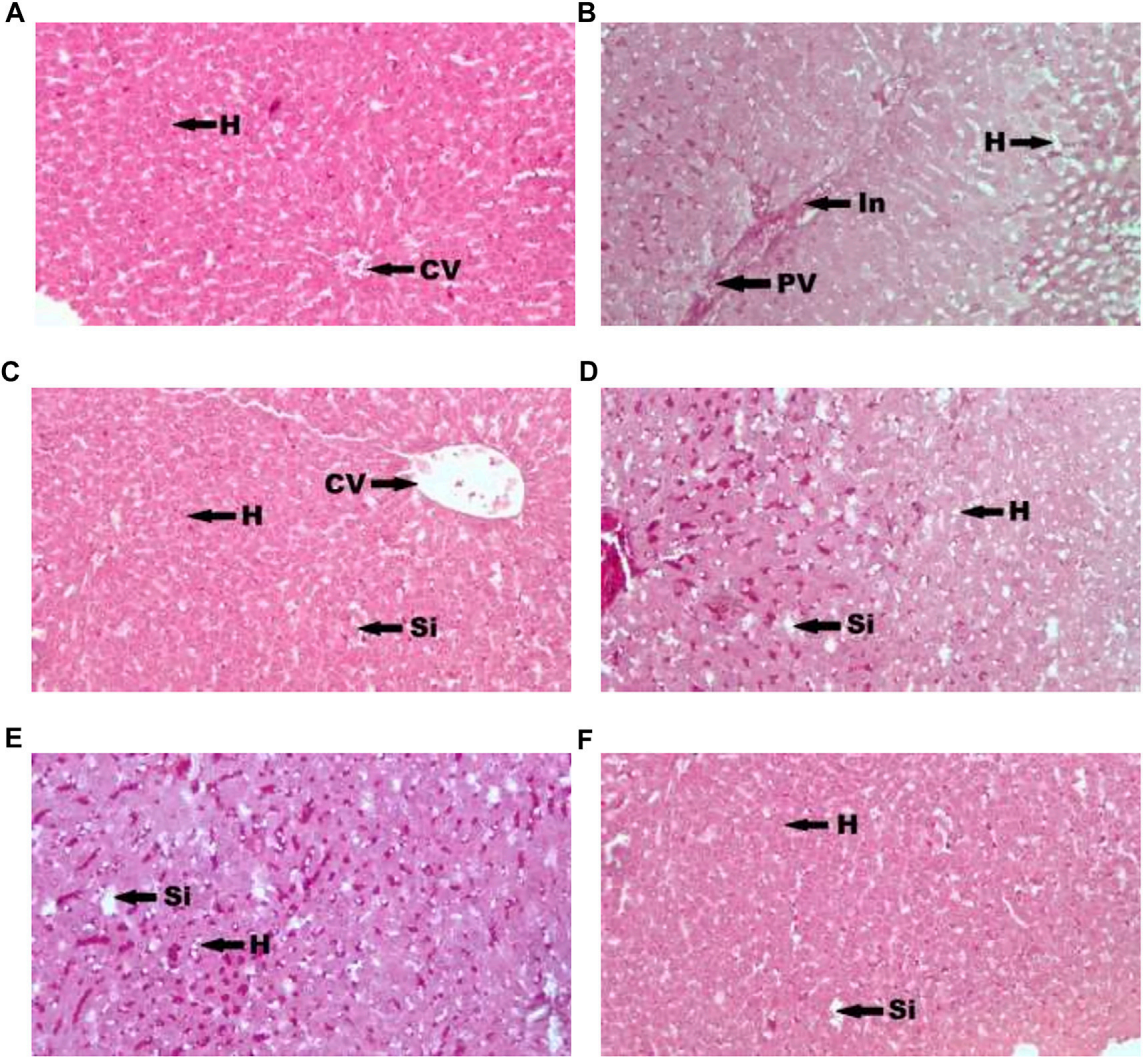
Photomicrograph of a section of liver of normal control **(A)**, diabetic control **(B)**, glibenclamide 20 mg/kg **(C)**, Spondias mombin 125 mg/kg **(D)**, Spondias mombin 250 mg/kg **(E)**, Spondias mombin 500 mg/kg **(F)**. H-hepatocytes, CV-centrilobular vein, PV-Portal vein, In-Periportal Inflammation, and Si-sinusoids. H&E 100X.

**FIGURE 3:**
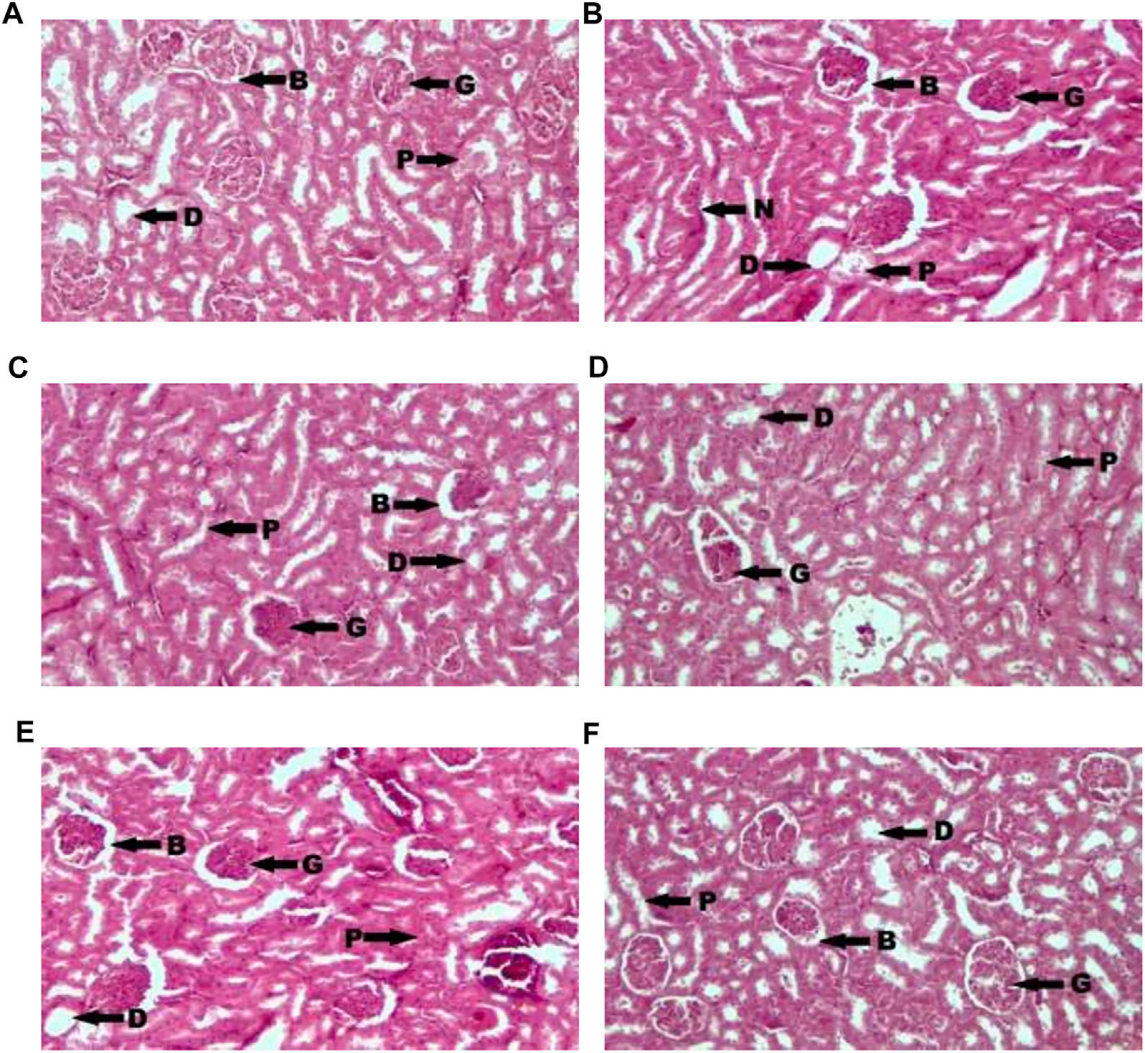
Photomicrograph of a section of kidney of normal control **(A)**, diabetic control **(B)**, glibenclamide 20 mg/kg **(C)**, Spondias mombin 125 mg/kg **(D)**, Spondias mombin 250 mg/kg **(E)**, Spondias mombin 500 mg/kg **(F)**. B-Bowman’s capsule, G-Glomerulus, P-Proximal convoluted tubule, D-Distal convoluted tubules, and N-Necrosis. H&E 100X.

Figure 1 showed photomicrograph of pancreas of normal control (A) rats showed normal architecture and the acinal cells are normal. Pancreas of diabetic control (B) rats showed degeneration of beta cells of Langerhans. Glibenclamide 20 mg/kg (C) treated rats showed regeneration of beta cells of Langerhans. Pancreas of *Spondias mombin* 125 mg/kg (D) treated rats showed minimal regeneration of beta cells of Langerhans. *Spondias mombin* 250 mg/kg (E) treated rats showed average regeneration of beta cells of Langerhans. However, *Spondias mombin* (500 mg/kg) treated rats showed pronounced regeneration of beta cells of Langerhans. Figure 2 exhibited photomicrograph of liver section of normal control (A) showing normal lobular pattern with a centrilobular vein and radiating irregular anastomosing plates of hepatocytes. Section of liver of diabetic control (B) showing accumulation of droplets with distorted morphology of hepatocytes, portal vein, and periportal inflammation. Section of liver of glibenclamide 20 mg/kg (C) treated rats showing restored morphology of hepatocytes, centrilobular vein and minimal dilation of sinusoids. *Spondias mombin* 125 mg/kg (D) treated rats showing moderated improvement in the slightly distorted hepatocytes and dilation of sinusoids. *Spondias mombin* 250 mg/kg (E) treated rats showing moderate improvement in the distorted hepatocytes and sinusoids. *Spondias mombin* 500 mg/kg (F) treated rats showing marked improvement in the distorted hepatocytes and sinusoids. Figure 3 Showed photomicrograph of a section of kidney of normal control (A) rats showing normal bowman’s capsule, glomerulus, and renal tubules. Kidney of diabetic control (B) rats showing distorted and shrunken glomerulus with proximal and distal convulated tubules and necrosis. Glibenclamide 20 mg/kg treated rats showing improved microarchitecture of bowman’s capsule, glomerulus and proximal and distal convulated tubules. *Spondias mombin* 125 mg/kg (D) treated rats showing minimum improvement in microarchitecture of bowman’s capsule, glomerulus and proximal and distal convulated tubules. *Spondias mombin* 250 mg/kg (E) treated rats showing moderate improvement in microarchitecture of bowman’s capsule, glomerulus and proximal and distal convulated tubules. Mild also necrosis observed. *Spondias mombin* 500 mg/kg (F) showed restored microarchitecture of bowman’s capsule, glomerulus, and proximal and distal convulated tubules.

## 4 Discussion

MESM showed no significant changes in the body weight throughout the study comparison to normal control rats. Oral administration of different doses of MESM caused significant decrease in glucose levels in STZ induced diabetic rats. Furthermore, our results revealed that diabetic rats treated with MESM for 28 days significantly recovered lipid and biochemical parameters within normal levels.

Administration of STZ was found capable of developing peripheral insulin resistance or impairing insulin secretion from pancreatic β cells and it is sufficient to induce noninsulin-dependent diabetes mellitus (type 2) in animals (Qinna and Badwan, 2015). STZ also causes diabetes by damaging beta-cells of the Islets of Langerhans in the pancreas due to uncontrolled production of reactive oxygen species (ROS) leads to decrease in insulin synthesis and release (Cerf, 2013). The purpose of STZ induced diabetic rat model used in this due to lineaments such as stable hyperglycemia without insulin requirement to survive and response to glibenclamide (Rabbani et al., 2010). Glibenclamide act as insulin secretogogues by closing K-ATP channels bypassing the β cell metabolism. Insulin secretion stimulated by increased blood glucose, free fatty acids, amino acids, gastrointestinal hormones (gastrin, cholecystokinin, secretin), gastric inhibitory peptide, sulfonylurea drugs (glyburide, tolbutamide), parasympathetic, β -adrenergic stimulation (Vaz et al., 2016).

Significant loss in body weight was observed in diabetic rats due to loss or damage of protein structure and muscle wasting (Fakhruddin et al., 2017). However, administration of MESM alleviated this condition almost comparable with glibenclamide treated rats agrees the idea of antidiabetic effect of MESM by recovering from muscle wasting.

MESM reduced blood glucose levels in diabetic rats which emphasizes extracts hypoglycaemic effect. Its antidiabetic effect could be due to the presence of free radical scavengers such as flavonoids, tannins and polyphenols reduce glucose absorption in small intestine, increase glucose update by peripheral tissues, enhance regulation of glycolysis process, and glycogen synthase (Adisakwattana, 2017). The possible mechanism of MESM on hypoglycemic action may be through potentiation of pancreatic secretion of insulin from β-cell of islets and/or due to enhanced transport of blood glucose to the peripheral tissue or by inhibition of endogenous glucose production or activation of gluconeogenesis in liver and muscles (Burcelin et al., 1995).

The declined plasma insulin in diabetic induced rats was reversed by oral administration of extract. This could be due to MESM’s ability to regenerate beta cells (Patel et al., 2012). Decrease in total protein, albumin, and glubulin levels in diabetic rats caused by clinical markers in diabetic nephrophaty such as elevated protein catabolism, proteinuria and albuminurea (Kaleem et al., 2008). Diabetic condition also lowered levels of albumin, globulin and A/G suggesting chronic liver infection. However, treatment with MESM elevated total protein, albumin, globulin, and A/G into normal by restoring liver function. This improved protein synthesis, prevented protein degradation and elevated insulin’s alanine, arginine, and glutamine uptake (Ramachandran et al., 2012). Furthermore, Serum levels like AST, ALT, and ALP increased in diabetic rats indicates liver damage and stimulated gluconeogenesis and ketogenesis. Toxicity action of STZ on liver showed secretion of liver cytosolic enzymes into bloodstream (Ghimire et al., 2018). Increase in levels of urea and creatinine indicated renal damage in diabetic rats due to insulin and glucose failed to improve gluconeogenesis process. Antioxidant nephroprotective role of flavonoids and polyphenol in MESM protected renal function during diabetes. Glucosides in extract might converted bilirubin into glucorunic, activated Constitutive Andostane Receptor (CAR) for bilirubin excretion and lowered total bilirubin levels to normalcy (Arthur et al., 2012). Our finding was supported by hitopathological analysis. STZ induced rats expressed cellular damage in pancreatic islets, liver histology, and renal glomeruli and tubules (Petchi et al., 2014). Rats treated with glibenclamide and MESM exhibited reduction in the pathological changes induced by STZ expressed protective effect of both glibenclamide and MESM.

Liver is important for detoxification of xenobiotics and liver damage related to exposure due excessive drugs leads to depletion in glutathione (GSH) levels plasma membrane damage, cellular necrosis and changes in serum lipids (Singh et al., 2016). Induction of diabetes altered lipoprotein and lipid profile in rats, increase in blood glucose levels was concomitant with changes in serum lipid indices in diabetic rats. These abnormalities caused by inactivated activity of lipolytic hormones on fat depots due to insulin action (Andallu et al., 2009). Inactivated lipoprotein lipase enzyme due to insulin deficiency during diabetic condition caused secondary complications from hypertryglyceridemia and hypercholesterolemia. HMG-CoA reductase is a rate limiting enzyme that metabolise high cholesterol LDL. However, insulin deficiency caused dyslipidemia due to inhibitory action of insulin on HMG-CoA reductase (Grice and Elmendorf, 2017). Diabetes-induced hyperlipidemia is responsible for excess movement of fat from adipose due to limited usage of glucose. Treatment with MESM reduced TC, TG, LDL, VLDL levels, and increased HDL levels suggesting that MESM possess possible hypolipidemic activity.

Flavonoids and tannins are important polyphenolic compounds which exhibited pharmacological activities during health problems especially oxidative stress, cardiovascular diseases, hyperglycemia cancers and inflammations (Zhang et al., 2015). Furthermore, flavonoids, tannins, saponins, and anthraquinone demonstrated primary antioxidant activity and useful in managing diabetic condition. Many research reports indicated that the natural antioxidants could inhibit biological enzymes such as Dipeptidyl Peptidase IV (DPP-4), α-amylase, and α- glycosidase which involved in diabetes mellitus type 2 (Deacon et al., 2000; Semighini et al., 2011). Hypoglycemic activity of MESM could be due to the presence of various phytochemical compounds acted individually or synergistically in reducing blood glucose by enhancing insulin production from pancreatic islets and glucose breakdown parallel with glibenclamide. Antioxiday assay indicates potent efficiency to DPPH, suggesting that polyphenolic compounds may be the main contributor to scavenging DPPH free radicals and polyphenolic compounds are the most efficient in reducing power in MESM.

## 5 Conclusion

Antidiabetic and antihyperlipidemic effect of methanolic extracts of *S. mombin* leaves is comparable with glibenclamide in term of its effect in reducing the elevated blood glucose levels and improved biochemical and lipid profile. Further studies, are required to investigate the phytoconstituents that responsible for antidiabetic effect *S. mombin* leaves.

## Data Availability

The original contributions presented in the study are included in the article/Supplementary Material, further inquiries can be directed to the corresponding author.
